# 2189 Scrambler therapy: Potential new treatment for central neuropathic pain?

**DOI:** 10.1017/cts.2018.181

**Published:** 2018-11-21

**Authors:** Maureen A. Mealy, Sharon L. Kozachik, Lawrence Cook, Marie T. Nolan, Thomas J. Smith, Michael Levy

**Affiliations:** 1 Johns Hopkins University School of Nursing; 2 University of Utah; 3 Johns Hopkins University School of Medicine

## Abstract

OBJECTIVES/SPECIFIC AIMS: Central neuropathic pain is a severely disabling consequence of conditions that cause tissue damage in the central nervous system (CNS) such as multiple sclerosis (MS) and neuromyelitis optica (NMO). It impacts mood, mobility and quality of life, but is often refractory to common treatments. Scrambler Therapy is an emerging non-invasive pain modifying technique that utilizes transcutaneous electrical stimulation of nociceptive fibers with the intent of re-organizing maladaptive signaling pathways. It has been examined for treatment of peripheral neuropathy with favorable safety and efficacy outcomes, but its use in central neuropathic pain has not been reported. We aim to explore acceptability and safety of Scrambler Therapy through a Phase II sham-controlled trial in NMO, and describe its use to date in central neuropathic pain. METHODS/STUDY POPULATION: Two patients with longstanding central neuropathic pain who failed multiple drug trials were treated as proof-of-concept, supporting the recent launch of a Phase II randomized controlled trial in NMO where patients receive 10 daily Scrambler treatments versus sham. Safety and acceptability from those recruited to date will be reported. Acceptability is measured by adherence and responses to patient surveys. RESULTS/ANTICIPATED RESULTS: We plan to recruit 22 patients, randomized 1:1 into experimental and sham arms. We will present acceptability and safety data for Scrambler use in patients with NMO who have been recruited by the time of this conference, as well as effectiveness data from two cases that have been completed outside of the trial. One case involved a 65-year-old woman with a 4-year history of central neuropathic pain following a C3-C5 TM. Her numerical rating scale (NRS) pain score was reduced to 0/10 from a baseline score of 5/10. The second case involved a 52-year-old woman with a 13-year history of pain following a medullary cavernoma bleed. Her baseline NRS pain score was 9/10, which was reduced to 0.5/10 post-treatment. No adverse events were reported. Pain relief was sustained at 30 days’ post-treatment. DISCUSSION/SIGNIFICANCE OF IMPACT: We are investigating the acceptability and efficacy of Scrambler Therapy for central neuropathic pain treatment in NMO. Proof-of-concept was supported by two patients whose pain scores improved considerably more in response to this treatment than with previous pharmacologic and non-pharmacologic interventions. Results from this trial may support future investigation in other disorders that cause damage in the CNS, including MS and TM.

